# Evaluating the therapeutic potential of idebenone and related quinone analogues in Leber hereditary optic neuropathy

**DOI:** 10.1016/j.mito.2017.01.004

**Published:** 2017-09

**Authors:** Patrick Yu-Wai-Man, Devorah Soiferman, David G. Moore, Florence Burté, Ann Saada

**Affiliations:** a Wellcome Trust Centre for Mitochondrial Research, Institute of Genetic Medicine, Newcastle University, Newcastle upon Tyne, UK; b Newcastle Eye Centre, Royal Victoria Infirmary, Newcastle upon Tyne, UK; c NIHR Biomedical Research Centre at Moorfields Eye Hospital and UCL Institute of Ophthalmology, London, UK; d Department of Clinical Neurosciences, School of Clinical Medicine, University of Cambridge, Cambridge, UK; e Monique and Jacques Roboh Department of Genetic Research, Hadassah-Hebrew University Medical Center, Jerusalem, Israel; f Department of Genetics and Metabolic Diseases, Hadassah-Hebrew University Medical Center, Jerusalem, Israel

**Keywords:** ATP, adenosine triphosphate, CCCP, carbonylcyanide-3 chlorophenylhydrazone, CoQ_1_, coenzyme Q_1_, CoQ_10_, coenzyme Q_10_, DCF, 2′,7′-dichlorodihydrofluorescein diacetate, DQ, decylubiquinone, GAL, restrictive, galactose medium, GLU, permissive, high-glucose medium, Idb, idebenone, LHON, Leber hereditary optic neuropathy, MB, methylene blue, MRS, magnetic resonance spectroscopy, mtDNA, mitochondrial DNA, MTG, MitoTracker Green, OCR, oxygen consumption rate, OXPHOS, oxidative phosphorylation, PARP, poly (ADP-ribose) polymerase, PBS, phosphate buffered saline, RCR, respiratory control ratio, RFU, relative fluorescent unit, RGC, retinal ganglion cell, ROS, reactive oxygen species, SEM, standard error of mean, TMRE, tetramethylrhodamine ethyl ester, Coenzyme Q_10_, Idebenone, Quinone, Leber hereditary optic neuropathy, Mitochondrial disease, Optic atrophy

## Abstract

Leber hereditary optic neuropathy (LHON) is an important cause of mitochondrial blindness among young adults. In this study, we investigated the potential of four quinone analogues (CoQ_1_, CoQ_10_, decylubiquinone and idebenone) in compensating for the deleterious effect of the m.11778G>A mitochondrial DNA mutation. The LHON fibroblast cell lines tested exhibited reduced cell growth, impaired mitochondrial bioenergetics and elevated levels of reactive oxygen species (ROS). Idebenone increased ATP production and reduced ROS levels, but the effect was partial and cell-specific. The remaining quinone analogues had variable effects and a negative impact on certain mitochondrial parameters was observed in some cell lines.

## Highlights

• Idebenone can partially compensate for the deleterious effect of the m.11778G > A mitochondrial DNA mutation.
• The beneficial effect of idebenone on mitochondrial function was only observed in a subgroup of patient-derived fibroblasts.
• Quinone analogues can have a detrimental effect on certain mitochondrial parameters when tested *in vitro*.

## 1 Introduction

Leber hereditary optic neuropathy (LHON) is a primary mitochondrial DNA (mtDNA) disorder characterised by bilateral sequential or simultaneous visual loss in young adults (Man et al., 2002). Affected patients develop a dense central scotoma and visual acuity deteriorates rapidly to 20/200 or worse. Three point mutations within the mitochondrial genome (m.3460G>A, m.11778G>A and m.14484T>C) account for about 90% of all cases, with the m.11778G>A mutation being by far the most prevalent (60–80%) cause of LHON worldwide (Newman and Biousse, 2004, Yu-Wai-Man et al., 2011). An abiding mystery of this disorder is why these mtDNA mutations have such a marked pathological predilection for retinal ganglion cells (RGCs) and their projecting axons within the optic nerve (Carelli et al., 2004). Spontaneous visual recovery has been reported in up to 25% of patients affected with LHON and the milder m.14484T>C mutation carries a relatively better visual prognosis (Newman and Biousse, 2004, Yu-Wai-Man et al., 2011). However, visual recovery is invariably incomplete and the majority of affected LHON carriers will remain severely visually impaired and classified as legally blind. The peak age of onset is in the second and third decades of life and the sudden onset of mostly irreversible visual loss in otherwise healthy young individuals has major socioeconomic consequences (Kirkman et al., 2009a). Treatment options for LHON remain limited and management is largely supportive, which is a frustrating situation for patients and their families, and the clinicians overseeing their care.

LHON has an estimated prevalence of 1 in 30,000 and there are considerable challenges, both practical and financial, in conducting adequately powered randomised controlled trials, which remain the gold standard for establishing the therapeutic efficacy and safety of a proposed intervention (Gorman et al., 2015, Man et al., 2003). Varying combinations of high-dose vitamins and supplements with putative mitochondrial antioxidant properties have been given to patients with LHON in the hope of improving the visual prognosis, but none of these drug cocktails are properly evidence-based (Pfeffer et al., 2013, Yu-Wai-Man et al., 2014). A promising class of compounds for LHON is the ubiquinone family of molecules, in particular idebenone. Most of the mtDNA mutations that are known to cause LHON affect complex I subunits and as a result, there is impaired transfer of high-energy electrons to complex III, which is an essential step in mitochondrial oxidative phosphorylation (OXPHOS) (Carelli et al., 2004). The structural and functional defect of complex I triggers a downstream cascade of events, which ultimately compromises RGCs and precipitate progressive optic nerve neurodegeneration. There is ongoing debate whether the bioenergetic deficit in LHON directly commits RGC to cell death or whether it is driven primarily by increased reactive oxygen species, or possibly both having a synergistic deleterious impact (Carelli et al., 2004, Levin, 2015, Yu-Wai-Man et al., 2011). Irrespective of this unresolved point of contention, an attractive dual strategy would be to improve electron transfer along the mitochondrial respiratory chain to maximise OXPHOS and ATP production, whilst minimising in parallel ROS levels.

Coenzyme Q_10_ (CoQ_10_) is natural lipid-soluble quinone analogue and as a result of its intrinsic hydrophobic properties, it freely circulates within the mitochondrial inner membrane (Hargreaves, 2014). The molecule contains a redox active benzoquinone ring that is conjugated to an isoprenoid side chain consisting of 10 isoprenyl units, with the actual number of these units forming the basis of the biochemical nomenclature. In its reduced form, CoQ_10_ is the predominant electron carrier of the mitochondrial respiratory chain and it mediates the efficient shuttling of electrons from complexes I and II, and other flavoprotein dehydrogenases, to complex III. As a result, oral supplementation of CoQ_10_ has been used for a broad range of mitochondrial OXPHOS diseases, but with the exception of patients with primary CoQ_10_ deficiency, there is no convincing evidence of any clear benefit (Hargreaves, 2014). A major limitation of CoQ_10_ is its inability to cross the blood-brain barrier and to rectify this biophysical constraint, newer-generation quinone analogues have been developed to increase the bioavailability of the active moiety and potentially maximise its therapeutic potential. One such compound is idebenone (2,3-dimethoxy-5-methyl-6-(10-hydroxydecyl)-1,4-benzoquinone), which has a shorter side chain and is a hydrosoluble molecule compared with CoQ_10_ (Imada et al., 1989, Nagai et al., 1989). Idebenone has shown promise as a treatment modality for patients with visual loss secondary to LHON, but only a subgroup of patients with the m.11778G>A mutation seems to benefit, and there is still uncertainty about the magnitude of the visual benefit when compared with the natural history of the disease (Carelli et al., 2011, Klopstock et al., 2013, Klopstock et al., 2011, Newman, 2011).

In order to obtain pre-clinical data and support the case for early-phase clinical trials, we have optimised an *in vitro* functional test panel to investigate the effects of candidate drug molecules on key aspects of mitochondrial function with the use of patient-derived primary fibroblasts (Golubitzky et al., 2011, Soiferman et al., 2014). As reported previously for patients with nuclear-encoded complex I respiratory chain disorders, such an experimental approach can provide not only new insights into disease mechanisms, but it is also an attractive, cost-effective approach for targeted drug screening. In this study, we have made use of primary fibroblast cell lines carrying the m.11778G>A mutation to firstly explore the pathological consequences of this mtDNA mutation on mitochondrial function and cell survival, and secondly, to investigate whether supplementation with various quinone analogues could potentially rescue (or alternatively exacerbate) the observed disease phenotype.

## 2 Materials and methods

### 2.1 Patients and fibroblast cell lines

Primary fibroblast cell cultures (LHON A-D) were established from skin biopsies obtained from four unrelated white Caucasian affected male LHON carriers harbouring the m.11778A>G mutation at homoplasmic levels (Table 1). All four patients presented with a classical pattern of visual loss characterised by bilateral sequential optic neuropathy and rapid painless visual deterioration to counting fingers (CF) or worse. None of them were treated with CoQ_10_, idebenone or other quinone analogues after a confirmed molecular diagnosis had been made and prior to a skin biopsy being taken. One patient (LHON-B) experienced a significant amount of visual recovery that was, however, limited to his right eye. Visual acuity in that eye improved from CF at the nadir to 20/30 about one year after first disease onset and in the absence of any specific treatment. All the skin biopsies were taken after the onset of visual loss with the patient's informed consent. This study had the relevant institutional approval and it complied with the Declaration of Helsinki.

**Table 1:** Clinical details of affected LHON carriers.

Patient	Onset (Yrs)^a^	Smoker^b^	Visual recovery^c^	Visual acuity^d^	
				RE	LE
LHON-A	21	No	No	HM	CF
LHON-B	18	No	Yes	20/30	CF
LHON-C	45	Yes	No	CF	CF
LHON-D	18	No	No	HM	HM

CF = counting fingers; HM = hand movements; LE = left eye; RE = right eye; Yrs = years.

a Age of onset of visual loss.

b Smoking status prior to disease onset.

c Spontaneous visual recovery with none of the patients having been treated with quinone analogues following disease onset.

d Best-corrected visual acuity at the last clinic visit, which was at a similar level at the nadir except for the right eye of patient LHON-B.

### 2.2 Materials

Aliquots of coenzyme Q_1_ (CoQ_1_), coenzyme Q_10_ (CoQ_10_), decylubiquinone (DQ, Sigma-Aldrich, Rehovot, Israel), and idebenone (Idb, Santhera Pharmaceuticals, Liestal, Switzerland) were kept frozen as 10 mM stock solutions in DMSO. MTG (Molecular Probes, Eugene, Oregon, USA), TMRE and DCF (Biotium, Harvard, CA, USA) were diluted and stored according to the manufacturer's instructions. Unless otherwise stated, reagents were obtained from Sigma-Aldrich (Rehovot, Israel).

### 2.3 Tissue culture and experimental conditions

Fibroblasts were maintained in permissive DMEM (Biological Industries, Kibbutz Beit Haemek, Israel) medium containing 4.5 g glucose (GLU) per liter and supplemented with 10% fetal calf serum, 50 μg/ml uridine, and 110 μg/ml pyruvate at 37 °C under 5% CO_2_ conditions. Cells were seeded in triplicates at a concentration of 4 × 103 cells/100 μl on four identical 96 well microtiter plates. The following day, the medium was removed and the wells were washed once with phosphate buffered saline (PBS) before the addition of fresh DMEM (GLU) medium or a restrictive glucose-free DMEM medium to prevent ATP production from glycolysis (Biological Industries, Kibbutz Beit Haemek, Israel) supplemented with 10% dialyzed fetal calf serum and 5 mM galactose (GAL). Quinone analogues at a final concentration of 1 μM were added to the growth medium as previously described (Golubitzky et al., 2011, Soiferman et al., 2014). Cell growth and mitochondrial function were assessed after a 72-hour incubation period.

### 2.4 Assessment of cell growth

Cell growth was assessed by measuring cellular content with a colorimetric method based on methylene blue (MB) staining of basophilic cellular components, which is independent of redox status (Pelletier et al., 1988). Cells in the microtiter wells were fixed with glutaraldehyde and stained with 1% MB in borate buffer, before being rinsed and dried. The absorbance of the extracted dye was measured at 620 nm (A620).

### 2.5 Intracellular ROS production

The growth medium was removed and replaced with 10 μM 2′,7′-dichlorodihydrofluorescein diacetate (DCF) in phosphate buffered saline with magnesium and calcium (PBS-MgCa) for 20 min at 37 °C under 5% CO_2_ conditions. DCF was replaced with 100 ml fresh PBS-MgCa, incubated at 30 °C for 20 min and fluorescence was measured subsequently with λ_ex_ 485 nm and λ_em_ 520 nm. ROS production was calculated as relative fluorescence units (RFU) normalized to cellular growth A620.

### 2.6 Cellular ATP content and mitochondrial ATP synthesis

Cellular ATP content was measured using the ATPlite (Perkin Elmer, Waltham, MA, USA) luminescence assay kit according to the manufacturer's instructions. For mitochondrial ATP synthesis, we adapted an assay that we have previously described in lymphocytes using microtiter plates (Negari et al., 2013). Briefly, cells were rinsed in PBS and incubated for 20 min at 37 °C in 50 μl assay buffer containing 150 mM KCl, 10 mM potassium phosphate buffer (pH 7.4), 25 mM Tris (pH 7.4), 2 mM EDTA, 0.025% fatty-acid-free bovine serum albumin (BSA), 40 μg/ml digitonin, 5 mM glutamate, and 1 mM malate. ATP content was calculated as relative luminescence (RLU) normalized to cellular growth A620.

### 2.7 Mitochondrial content and membrane potential

Cells grown in microtiter plates were incubated with 200 nM MitoTracker Green (MTG) in growth medium for 45 min at 37 °C under 5% CO_2_ conditions before the addition of 50 nM tetramethylrhodamine ethyl ester (TMRE) for an additional 45 min. The medium was removed and after being rinsed, the cells were replaced in PBS. Fluorescence was measured at 37 °C with λ_ex_ 485 nm and λ_em_ 528 nm for MTG, and λ_ex_ 485 nm and λ_em_590 nm for TMRE. Mitochondrial content was calculated as MTG RFU normalized to cell growth A620. Mitochondrial membrane potential (Δψm) was derived from the relative fluorescence ratio of TMRE to MTG (TMRE-RFU/MTG-RFU). All luminescence, fluorescence and absorbance measurements in microtiter wells were performed with a Synergy HT microplate reader (Bio-Tek instruments, Vinoosky VT, USA).

### 2.8 Oxygen consumption

Oxygen consumption rate (OCR) was measured using an XF24 extracellular flux analyzer (Seahorse Biosciences, North Billeric, MA, USA). Fibroblasts were seeded 20,000 cells/well on an XF24-well plate in 0.3 ml GLU medium. The following day cells were rinsed once with PBS and medium was replaced with 0.5 ml fresh GLU, with or without quinone analogues at a final concentration of 1 μM. After 72 h, the growth medium was changed to a medium with the same composition as GLU, but with unbuffered DMEM (Seahorse Biosciences, North Billeric, MA, USA) and the plate was equilibrated at 37 °C for 1 h before the measurements. After 10 min of basal OCR measurements, carbonylcyanide-3 chlorophenylhydrazone (CCCP) was injected to reach a final concentration of 5 μM and the maximal OCR was measured. Background OCR was measured after injection of rotenone and antimycin to a final concentration of 3 μM each. OCR was normalized to cellular growth. Respiratory control ratio (RCR) was estimated as maximal/basal OCR ratio.

### 2.9 Apoptosis assays

Apoptosis was assessed by western blot analysis of two markers, caspase 3 and poly (ADP-ribose) polymerase (PARP). Cells treated with staurosporine (Merck Millipore) were included as a positive control group. Total protein concentration was determined with the Bradford assay. Cell lysates containing 30 μg of protein were loaded onto pre-cast NuPAGE 4–12% Bis-Tris gels (Life Technologies) and electrophoresis was carried out at 200 V in NuPage MES running Buffer (Life Technologies). Gels were transferred to PVDF membranes using iBlot Gel Transfer Stacks (Life Technologies) and membranes were probed with anti-caspase 3 (1 in 500, Cell Signaling, #9662), anti-PARP (1 in 500, Cell Signaling, #9542), and anti-β tubulin (1 in 2000, Abcam, #15568) antibodies. Following incubation with horseradish peroxidase-conjugated secondary antibodies (1 in 2000, Dako, P0399), signals were visualised with Clarity Western ECL substrate (Bio-Rad) and the Biospectrum 500 Imaging System (UVP). The results obtained were normalized to β tubulin.

For the TUNEL assay, cells were grown in 96-well microtiter plates in restrictive galactose medium for 72 h. Apoptosis was assessed by a quantitative colorimetric assay using the HT TiterTACS assay kit (Trevigen, Gaithersburg, MD, USA) as per the manufacturer's protocol.

### 2.10 Statistical analysis

The data are shown as the mean ± standard error of the mean (SEM) of triplicate independent experiments. Groups were compared using the Student's *t*-test with the IBM-SPSS™ v.20 software (IBM Corp. Armonk, NY) and a *P* value < 0.05 was considered as statistically significant.

## 3 Results

### 3.1 LHON fibroblasts exhibit decreased cell growth

**Fig. 1:**
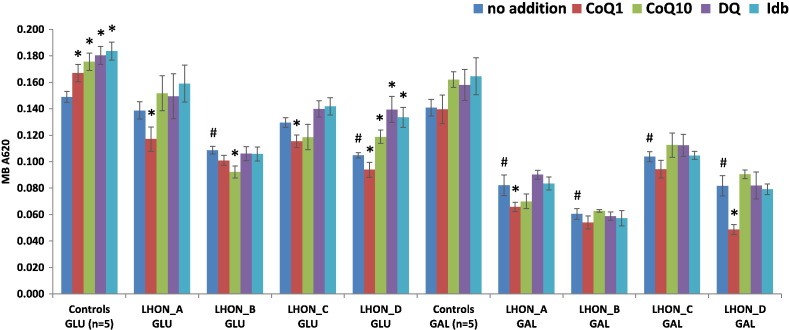
Effect of culture media and quinone analogues on cell growth. Cells were grown either in permissive glucose-containing medium (GLU) or in restrictive galactose (GAL) medium. Cells were also supplemented with 1 μM quinone analogues; coenzyme Q_1_ (CoQ1), coenzyme Q_10_ (CoQ10), decylubiquinone (DQ) or idebenone (Idb). Cell growth was measured by methylene blue (MB A620) in either GLU or GAL medium. The results are presented as the mean ± SEM (^#^*P* < 0.05 effect of m.11778G>A compared with controls (*n* = 5) in the same medium; *P < 0.05 effect of additive).

Cell growth was reduced in the LHON cell lines compared with controls in permissive glucose-containing media and this effect was significant for LHON-B and LHON-D (Fig. 1). The impairment in cell growth caused by the m.11778G>A mutation was further accentuated in restrictive galactose media, with all four LHON cell lines showing a marked and significant decrease compared with glucose media. There was a trend towards improved cell growth with CoQ_10_, decylubiquinone and idebenone in most cells, which was significant for controls and LHON-D under glucose, but not galactose media conditions. Paradoxically, CoQ_1_ had a significant detrimental effect on cell growth when added to three of the four LHON cell lines (LHON-A, LHON-C and LHON-D).

### 3.2 ROS production is increased in LHON fibroblasts

**Fig. 2:**
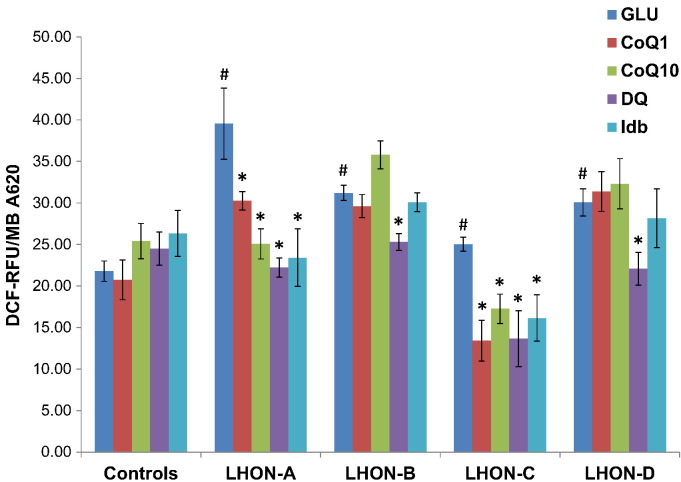
Effect of quinone analogues on ROS production. Cells were grown in glucose-containing medium (GLU) only or in the presence of 1 μM quinone analogues; coenzyme Q_1_ (CoQ1), coenzyme Q_10_ (CoQ10), decylubiquinone (DQ) or idebenone (Idb). The level of ROS production was assayed by DCF. The fluorescence (DCF-RFU) was normalized to cell growth (MB A620) and the results are presented as the mean ± SEM (^#^*P* < 0.05 effect of m.11778G>A compared with controls (*n* = 5); *P < 0.05 effect of additive).

All four LHON cell lines demonstrated significantly elevated ROS levels compared with controls, especially LHON-A (Fig. 2). A marked reduction in ROS levels was observed for LHON-A and LHON-C with all four quinone analogues tested. Decylubiquinone was the most effective agent with ROS production decreasing to control levels or lower in all four LHON cell lines. LHON-B and LHON-D did not show a positive response to CoQ_1_ and idebenone, and there was a trend towards increased ROS levels in LHON-B with CoQ_10_ supplementation, which was not statistically significant.

### 3.3 Mitochondrial bioenergetics is impaired by the m.11778G>A mtDNA mutation

**Fig. 3:**
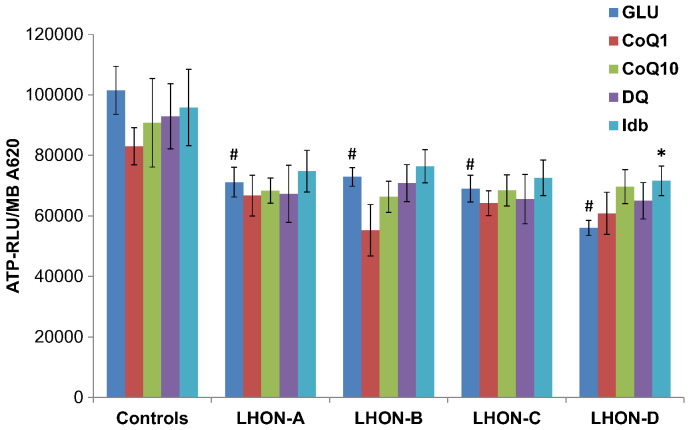
Mitochondrial ATP production. Cells were grown in glucose-containing medium only (GLU) or in the presence of 1 μM quinone analogues; coenzyme Q_1_ (CoQ1), coenzyme Q_10_ (CoQ10), decylubiquinone (DQ) or idebenone (Idb). Mitochondrial ATP content was measured after digitonin treatment and incubation with glutamate and malate. The relative luminescence (RLU) was normalized to cell growth (MB A620) and the results are presented as the mean ± SEM (^#^*P* < 0.05 effect of m.11778G>A compared with controls (*n* = 5); *P < 0.05 effect of additive).

Mitochondrial membrane potential was not adversely affected by the m.11778G>A mutation or influenced by the quinone additives, except for LHON-C where a relative decrease compared with the untreated values for this cell line was observed with CoQ_1_ (Supplementary Fig. 1). Although cellular ATP content was not compromised (data not shown), mitochondrial ATP synthesis was markedly reduced in all four LHON cell lines. No significant improvement in mitochondrial ATP synthesis was observed on supplementation with quinone analogues, except for the LHON-D cell line when treated with idebenone (Fig. 3).

**Fig. 4:**
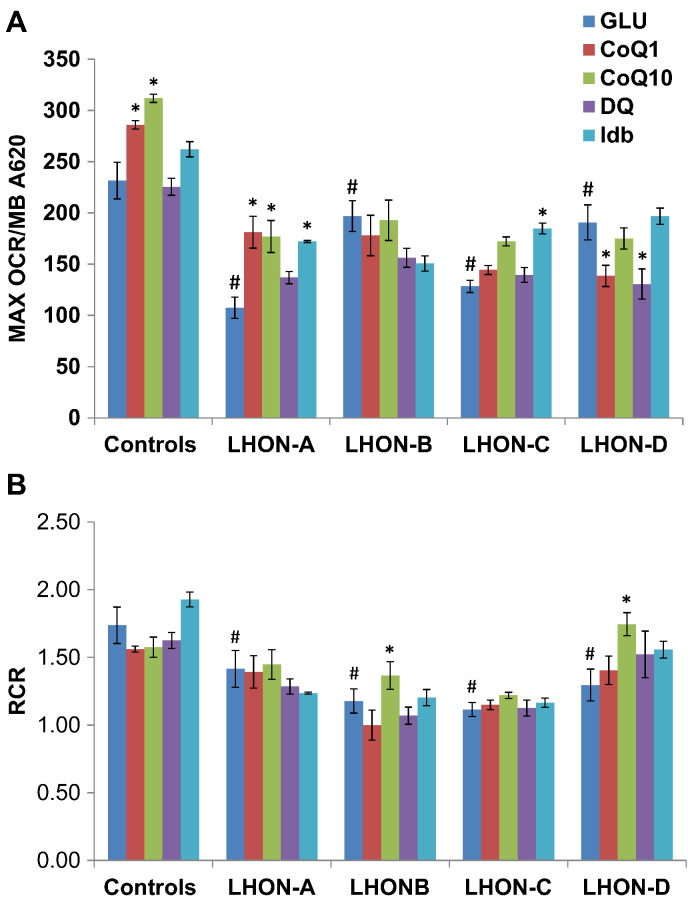
Mitochondrial respiration under basal and maximal conditions. Oxygen consumption was measured in cells grown in glucose-containing medium only (GLU) or in the presence of 1 μM quinone analogues; coenzyme Q_1_ (CoQ1), coenzyme Q_10_ (CoQ10), decylubiquinone (DQ) or idebenone (Idb). [A] Maximal oxygen consumption rate (MAX OCR) after the addition of an uncoupler CCCP and normalized to cell growth (MB A620). [B] Respiratory control ratio (RCR) calculated as the ratio between MAX OCR and the basal oxygen consumption rate. The results are presented as the mean ± SEM (*P < 0.05 effect of m.11778G>A compared with controls (*n* = 5); ^∧^* P* < 0.05 effect of additive).

Maximal OCR was decreased for all four LHON cell lines and this was particularly pronounced for LHON-A and LHON-C (Fig. 4A). A partial decrease in RCR, which is a marker of mitochondrial uncoupling, was also observed (Fig. 4B). Idebenone had a significant positive effect on maximal OCR in LHON-A and LHON-C with RCR remaining unchanged. The remaining quinone analogues demonstrated variable effects. Interestingly, CoQ_1_ and decylubiquinone decreased maximal OCR in LHON-D without significantly affecting RCR.

### 3.4 No evidence of apoptosis in LHON fibroblasts

Western blot analysis of cleaved caspase-3 and PARP showed no significant difference between all four LHON cell lines when compared with controls (Supplementary Fig. 2). Similarly, there was no evidence of increased apoptosis with the TUNEL assay under galactose media conditions (data not shown).

## 4 Discussion

The m.11778G>A mutation accounts for the majority of LHON cases worldwide and it affects a key complex I subunit of the mitochondrial respiratory chain (Newman and Biousse, 2004, Yu-Wai-Man et al., 2011). There is currently some debate in the literature on the pathophysiological mechanisms that link mtDNA LHON mutations with RGC loss. Previous work on LHON cybrids and a variety of patient-derived tissues, including blood and skeletal muscle, indicate impairment in mitochondrial biogenesis with reduced complex I-driven ATP synthesis (Carelli et al., 2004, Yu-Wai-Man et al., 2011). This observation has been further substantiated *in vivo* with ^31^P–MRS (magnetic resonance spectroscopy) and the m.11778G>A mutation was associated with the most pronounced reduction in mitochondrial ATP synthesis, followed by the m.14484T>C, and m.3460G>A mutation (Lodi et al., 1997). Besides the impact of LHON mutations on OXPHOS, there is increasing awareness of the potential primary role of increased ROS levels in triggering RGC loss (Levin, 2015). A mouse model has been created with a homoplasmic m.13997G>A mutation within *MTND6*, which causes an amino acid substitution (p.Pro25Leu) similar to the m.14600G>A mutation previously reported in a family with Leigh syndrome and optic atrophy (Malfatti et al., 2007). The mutant mice developed some of the key histopathological features seen in the optic nerves of affected LHON patients, namely, neuronal accumulation of abnormal mitochondria, swelling of RGC axons and demyelination (Lin et al., 2012). Interestingly, mitochondrial analysis showed decreased complex I activity and increased ROS production, but no diminution of ATP production was detected.

Our data obtained from patient-derived fibroblasts harbouring the m.11778G>A mutation suggest that both impaired mitochondrial bioenergetics and elevated cellular ROS levels could contribute to RGC loss in LHON, perhaps in a synergistic fashion as both factors are intrinsically related. Strikingly, cell growth was markedly impaired in all four LHON cell lines especially when they were deprived of glucose under restrictive galactose media conditions, thereby forcing them to rely solely on mitochondrial OXPHOS as their energy source. Accordingly, mitochondrial ATP production was also markedly decreased whilst ROS production was elevated. Although we did not find evidence of increased apoptosis, this might simply reflect the limitation of the cell type that was used in our experiments. More plausibly, RGCs carrying a pathological LHON mutation operate under a precarious environment within the inner retina, dominated by chronic mitochondrial dysfunction, until a tipping point is reached that precipitates disease conversion in at-risk LHON carriers. These more dynamic situations in a complex neuronal structure like RGCs are clearly not reproducible in a simpler *in vitro* fibroblast model. In keeping with the need for a secondary trigger, recent studies have reinforced the role of smoking and a reduction in circulating oestrogen levels as risk factors for disease conversion in LHON (Giordano et al., 2011, Giordano et al., 2015, Kirkman et al., 2009b).

Spontaneous visual recovery in LHON, when it does occur, is incomplete and the majority of patients remain chronically visually disabled (Kirkman et al., 2009a). Treatment options for this mitochondrial disorder are still limited, but pharmacological options aimed at improving mitochondrial OXPHOS and reducing ROS levels are offering renewed hope to patients and their families. There are several limitations to the use of CoQ_10_ in LHON, in particular its inability to cross the blood-brain barrier in order to reach sufficiently high levels within the RGC layer (Giorgio et al., 2012, Gueyen et al., 2015, Hargreaves, 2014). Idebenone is a newer-generation quinone analogue that circumvents this therapeutic barrier and based on anecdotal LHON case reports, this compound seemed to have a degree of visual benefit for some patients, but not all (Barnils et al., 2007, Haefeli et al., 2011, Heitz et al., 2012, Mashima et al., 2000). Those initial observations led to the RHODOS (Rescue of Hereditary Optic Disease Outpatient Study, ClinicalTrials.gov identifier: NCT00747487) trial, which was a multicentre, double-blind, randomised treatment study comparing idebenone, at a dose of 300 mg three times per day, with placebo (Klopstock et al., 2011). A total of 85 patients with a confirmed mtDNA mutation (m.3460G>A, m.11778G>A, and m.14484T>C) and with disease duration of up to five years were recruited. The RHODOS trial failed to show a benefit for its pre-specified primary end point (best recovery of visual acuity at week 24), but all of the secondary end points showed a positive trend towards visual improvement in the idebenone-treated group (Klopstock et al., 2011). A retrospective Italian study of 103 patients subsequently indicated that patients receiving idebenone were more likely to recover vision if treatment was initiated early and if it was maintained for longer than the 24-week regimen used in the RHODOS trial (Carelli et al., 2011). A marketing authorisation application by Santhera Pharmaceuticals Ltd. (Liestal, Switzerland) to the European Medicines Agency's Committee for Medicinal Products for Human Use (CHMP) was initially rejected, but based on additional data collected as part of a named-patient access programme, idebenone has recently been approved under exceptional circumstances for the treatment of visual impairment in patients affected with LHON (http://www.ema.europa.eu/ema/index.jsp?curl=pages/medicines/human/medicines/003834/smops/Positive/human_smop_000835.jsp&mid=WC0b01ac058001d127, accessed on 28 December 2016). Overall, the current body of evidence does support a visual benefit with idebenone in acute LHON, but only in a subgroup of treated patients and importantly, it is not possible to predict who will respond. Furthermore, it should be stressed that there is no solid evidence base to guide the optimal dose and duration of treatment, and it remains debatable whether idebenone has any beneficial effect once optic atrophy has become established in the chronic phase of the disease.

Our study on LHON fibroblasts confirms that idebenone can have a positive effect on OXPHOS by promoting the efficiency of electron flux along the mitochondrial respiratory chain. This effect was associated in two cell lines (LHON-A and LHON-C) with a consequent reduction in ROS production and in one cell line (LHON-D) with improved ATP production. Importantly, the response to quinone supplementation was cell-specific and the detrimental effects of the m.11778G>A mutation on mitochondrial function were not fully compensated. The partial response with idebenone supplementation and the observed variability between the LHON cell lines essentially mirrors the clinical experience of treating patients with idebenone (Carelli et al., 2011, Klopstock et al., 2013, Klopstock et al., 2011, Newman, 2011). A note of caution on the use of certain quinone analogues for patients with LHON, and by extension other mitochondrial diseases, is also warranted. It is well established that drugs can have marked inter-individual responses and the assumption of safety for a particular class of molecules does not preclude idiosyncratic adverse effects. In the LHON cell lines used in our study, certain mitochondrial parameters were adversely affected by CoQ_1_, CoQ_10_ and decylubiquinone, with the detrimental effect being more pronounced with CoQ_1._ Although, we did not observe a generalised detrimental response with any of these compounds, and with the added caveat that we only tested four LHON cell lines, all of which carried the m.11778G>A mutation, these findings do highlight the central concept of *primum non nocere* in medical practice. CoQ_1_ has been found to impair astrocyte function, possibly through the depletion of NAD(P)H (Dragan et al., 2006). Although idebenone did not have any negative effects on the cells and parameters used in our study, rather surprisingly, idebenone had an adverse impact on RGC dendrite morphology and visual function when fed to wild-type mice (Smith et al., 2016). We still have an incomplete understanding of the pathophysiology of LHON and the variable modes of action of different quinone analogues, including idebenone, clearly substantiate the conservative approach adopted by most experts not to recommend their prophylactic use for asymptomatic LHON carriers. The therapeutic benefit of other shorter-chain quinone analogues such as alpha-tocotrienol quinone (EPI-743), which can cross the blood brain barrier, also need to be investigated further (Shrader et al., 2011).

## 5 Conclusions

Consistent with recently published clinical studies, our *in vitro* fibroblast data indicate a patient-specific response to idebenone and this specific quinone analogue is not able to fully reverse the deleterious consequences of the m.11778G>A mutation on mitochondrial function. Further work is urgently needed to develop better treatment options for this devastating cause of mitochondrial blindness among young adults.

The following are the supplementary data related to this article.

Supplementary Fig. 1 Mitochondrial membrane potential in LHON and control fibroblasts. Cells were grown in glucose-containing medium only (GLU) or in the presence of 1 μM quinone analogues; coenzyme Q_1_ (CoQ1), coenzyme Q_10_ (CoQ10), decylubiquinone (DQ) or idebenone (Idb). Mitochondrial membrane potential (MMP) was measured by TMRE (TMRE-RFU) and normalized to mitochondrial content measured by MitoTracker Green (MTG-RFU). The results are presented as the mean ± SEM (^#^*P* < 0.05 effect of m.11778G > A compared with controls (*n* = 5); *P < 0.05 effect of additive). Supplementary Fig. 1

Supplementary Fig. 2 Western blot analysis of caspase-3 and cleaved PARP. Representative western blot of caspase-3 and PARP protein levels in LHON and control fibroblasts grown under galactose media conditions only or in the presence of idebenone (Idb). Supplementary Fig. 2

## Financial disclosures

PYWM holds a consultancy agreement with GenSight Biologics (Paris, France). No other relevant financial disclosures or conflicts of interest.
