# Supplementary figures and images for: Evaluating the therapeutic potential of idebenone and related quinone analogues in Leber hereditary optic neuropathy
Source: Mitochondrion. 2017 Sep;36:36–42. doi: 10.1016/j.mito.2017.01.004 (PMC5644719; doi:10.1016/j.mito.2017.01.004)

## Slide 1
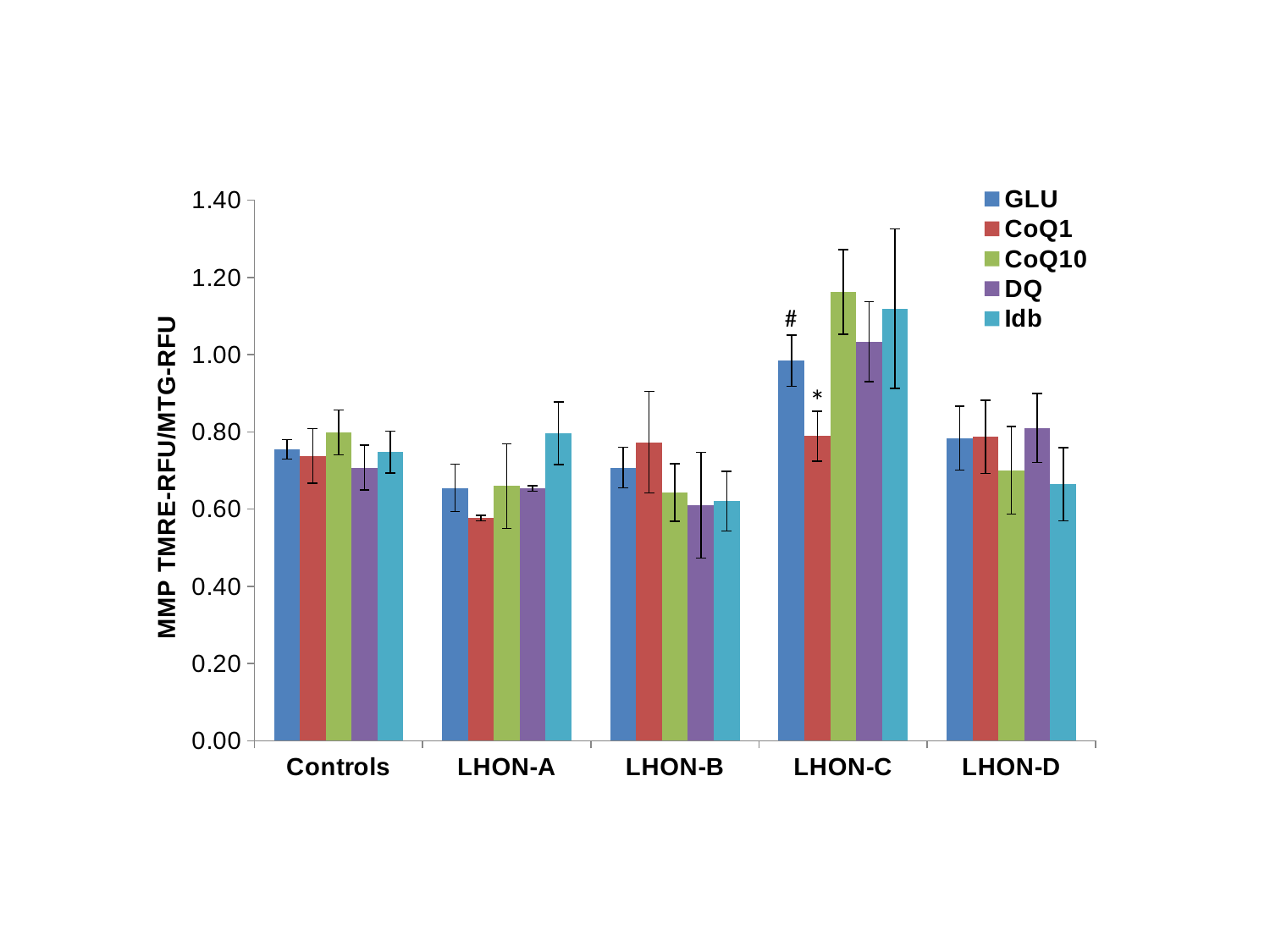

### Chart
| Category | GLU | CoQ1 | CoQ10 | DQ | Idb |
|---|---|---|---|---|---|
| Controls | 0.7553377640926512 | 0.7381141545574355 | 0.7987286382229134 | 0.7074426999284836 | 0.7477491855911483 |
| LHON-A | 0.654844111469315 | 0.5766878508840969 | 0.6596537172742076 | 0.6532375729523043 | 0.7963573668714798 |
| LHON-B | 0.707425652639631 | 0.7732674543085962 | 0.642564626863175 | 0.6107065965838003 | 0.6209658394657289 |
| LHON-C | 0.9844113982650123 | 0.789095820158658 | 1.1622702679488202 | 1.0336932168658424 | 1.1192852452050932 |
| LHON-D | 0.7841206264276493 | 0.7875435142909498 | 0.7002970869084688 | 0.8101603894594462 | 0.6641924438188824 |#
*

Supplement: Supplementary Fig. 1 — Mitochondrial membrane potential in LHON and control fibroblasts. Cells were grown in glucose-containing medium only (GLU) or in the presence of 1 μM quinone analogues; coenzyme Q1 (CoQ1), coenzyme Q10 (CoQ10), decylubiquinone (DQ) or idebenone (Idb). Mitochondrial membrane potential (MMP) was measured by TMRE (TMRE-RFU) and normalized to mitochondrial content measured by MitoTracker Green (MTG-RFU). The results are presented as the mean ± SEM (#P < 0.05 effect of m.11778G > A compared with controls (n = 5); *P < 0.05 effect of additive). [file mmc1.pptx]
