# Supplementary material for: Evaluating the therapeutic potential of idebenone and related quinone analogues in Leber hereditary optic neuropathy
Source: Mitochondrion. 2017 Sep;36:36–42. doi: 10.1016/j.mito.2017.01.004 (PMC5644719; doi:10.1016/j.mito.2017.01.004)
Supplement: Supplementary Fig. 2 — Western blot analysis of caspase-3 and cleaved PARP. Representative western blot of caspase-3 and PARP protein levels in LHON and control fibroblasts grown under galactose media conditions only or in the presence of idebenone (Idb). [file mmc2.pptx]

## Slide 1
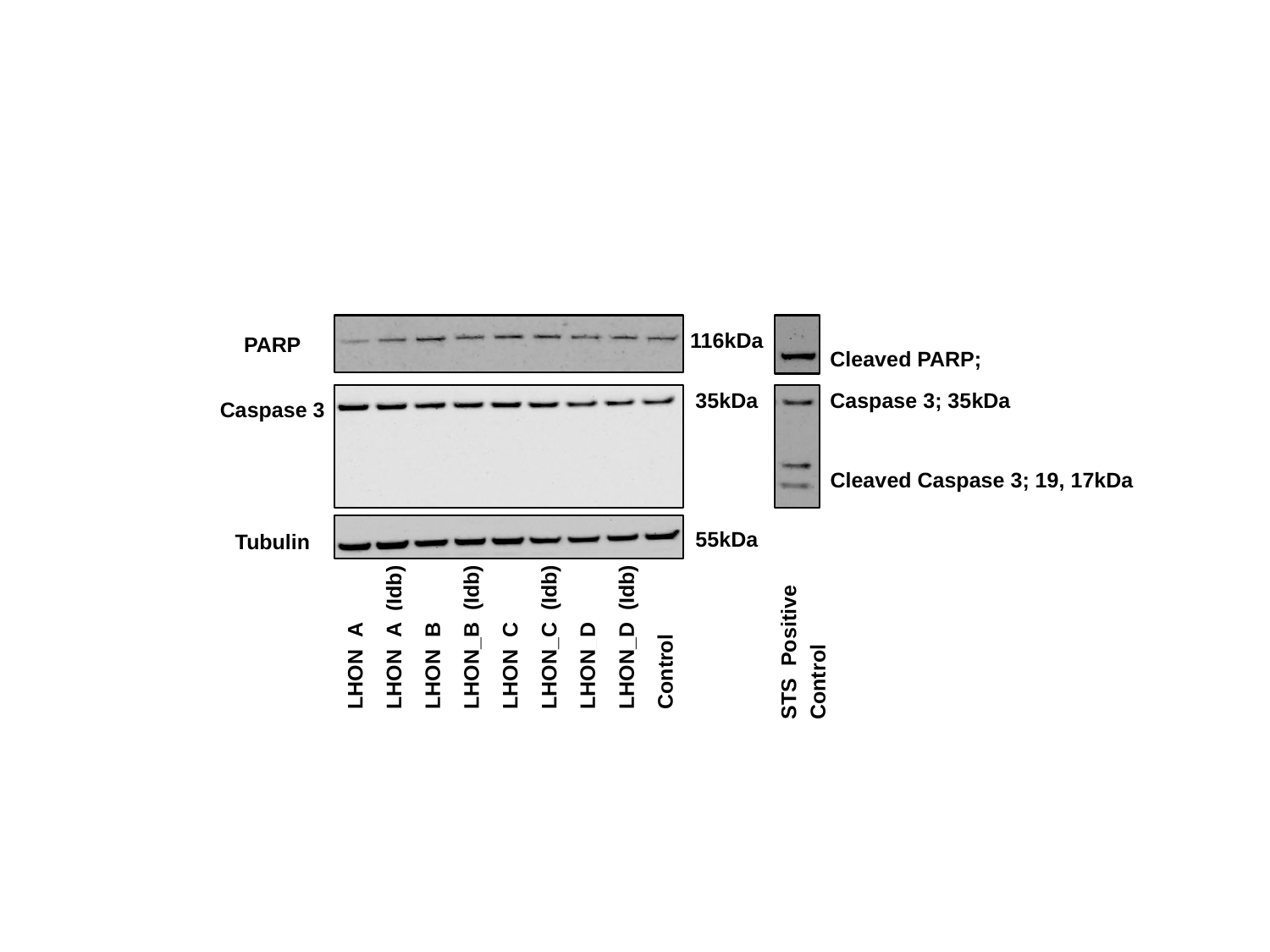

116kDa
PARP
Cleaved PARP; 89kDa
35kDa
Caspase 3; 35kDa
Caspase 3
Cleaved Caspase 3; 19, 17kDa
55kDa
Tubulin
STS Positive Control
LHON_C (Idb)
LHON_D (Idb)
LHON_A (Idb)
LHON_B (Idb)
LHON_A
LHON_D
LHON_B
LHON_C
Control
